# A Global User-Driven Model for Tile Prefetching in Web Geographical Information Systems

**DOI:** 10.1371/journal.pone.0170195

**Published:** 2017-01-13

**Authors:** Shaoming Pan, Yanwen Chong, Hang Zhang, Xicheng Tan

**Affiliations:** 1 State Key Laboratory of Information Engineering in Surveying, Mapping and Remote Sensing, Wuhan University, Wuhan, Hubei, China; 2 School of Remote Sensing and Information Engineering, Wuhan, Hubei, China; 3 International School of Software, Wuhan University, Wuhan, Hubei, China; 4 Engineering Research Center for Geo-Informatics and Digital Technology Authorized by National Administration of Surveying, Mapping and Geoinformation, Wuhan University, Wuhan, Hubei, China; West Virginia University, UNITED STATES

## Abstract

A web geographical information system is a typical service-intensive application. Tile prefetching and cache replacement can improve cache hit ratios by proactively fetching tiles from storage and replacing the appropriate tiles from the high-speed cache buffer without waiting for a client’s requests, which reduces disk latency and improves system access performance. Most popular prefetching strategies consider only the relative tile popularities to predict which tile should be prefetched or consider only a single individual user's access behavior to determine which neighbor tiles need to be prefetched. Some studies show that comprehensively considering all users’ access behaviors and all tiles’ relationships in the prediction process can achieve more significant improvements. Thus, this work proposes a new global user-driven model for tile prefetching and cache replacement. First, based on all users’ access behaviors, a type of expression method for tile correlation is designed and implemented. Then, a conditional prefetching probability can be computed based on the proposed correlation expression mode. Thus, some tiles to be prefetched can be found by computing and comparing the conditional prefetching probability from the uncached tiles set and, similarly, some replacement tiles can be found in the cache buffer according to multi-step prefetching. Finally, some experiments are provided comparing the proposed model with other global user-driven models, other single user-driven models, and other client-side prefetching strategies. The results show that the proposed model can achieve a prefetching hit rate in approximately 10.6% ~ 110.5% higher than the compared methods.

## Introduction

Similar to most of time-based tasks, reducing resource consumption and improving the response speed are two key problems for a web geographical information system (GIS). Thus, many methods have been proposed to improve system performance on-the-fly, including PGSW-OS [1], MR-D [2], 2D-WDM [3], and LLLA [4], among others. Among these methods, PGSW-OS uses P2P (peer-to-peer) nodes to share resources to reduce the total resource consumption of the system. MR-D also uses a type of device-to-device (D2D) network to share the cellular spectrum. 2D-WDM is designed to improve network transmission efficiency by considering not only the time but also the wavelength in a wavelength-division multiplexed (WDM) system. LLLA provides a new method to improve the efficiency of channel assignment in wireless mesh networks(WMNs). Obviously, all these methods can be profitably applied to a system to reduce resource consumption and improve response speed. However, compared to improving the transmission efficiency of the system by optimizing network topology, improving channel efficiency or capacity and preparing data for servers (or users) in advance can also be employed to reduce the response delay due to slow disk I/O speeds.

In fact, prefetching and caching is one pair of fundamental techniques for improving the quality of service and reducing the response time for users in web GIS. In this model, the data are split into smaller chunks called tiles [5] based on the pyramid model. A web GIS is a typical service-intensive application [6] that handles large numbers of user requests at the same time. The main purpose of a prefetching and caching model in GIS is to prefetch "hotspot" tiles from normal storage and put them into a high-speed cache pool, thus, reducing the request-response time and, ultimately, improving the system's performance. Because a server has a limited amount of high-speed cache space as well as a large number of tiles that need to be cached based on the large number of user requests, determining which tiles to prefetch and cache is difficult. Consequently, numerous related studies have been performed to address these key problems.

Some traditional classic algorithms such as FIFO (first in first out) and LRU (least recently used) are widely used by Google [7], NASA [8] and networked geographic information systems (NGISs) [9], and all these have achieved some good results. However, there are two main problems with these types of traditional algorithms: 1) FIFO and LRU consider only cache replacement policies; they do not address the problem how best to proactively prefetch tiles from storage to prepare data for users in advance; and 2) they use only the information inherent to the data itself to determine whether a certain tile should be replaced; they do not consider the fact that some very hot tiles will be accessed repeatedly and some related tiles exist that will be accessed simultaneously. These hot tiles should be kept in the cache buffer constantly rather than to replaced.

In fact, access to tiles satisfies some intrinsic laws. One example is Zipf-like laws, which indicate that the demand for tiles is extremely unbalanced: some 20% of the requested tiles may receive 80% of the total number of requests [10,11]. Another example is the spatial locality principle which shows the relationships among all tiles [12]. Such a priori knowledge can be used to find tiles that will be requested simultaneously and immediately (i.e., over a short period of time) after a certain tile is requested and then prefetch them proactively. It can be also used to determine which tiles those in the high-speed cache buffer will not be requested within a short time period and replace them to save cache space [13,14].

Obviously, all these intrinsic laws are driven by user access behaviors; therefore, knowledge of such behaviors can be used to optimize system performance [15]. The main idea behind this method, called a "user-driven" model (some researchers term this a trace-driven model [16]), is to find the relationships among all tiles according to historical access-log information (the logged data are called traces and are accumulated by GISs after long periods of use) and use that relationship information to determine which tiles should be prefetched or replaced based on their relationship with the next requested tile.

Many prefetching and cache replacement strategies based on user-driven models exist—each with its own algorithms for selecting the objects to be prefetched or replaced. Examples of such strategies include both client-side driven models that prefetch tiles from the client and server-side driven models that prefetch tiles from the server. For example, Retrospective Adaptive Prefetch (RAP) [17] is a client-side driven model, while Tile Prefetching Based on Previous *k* Movements (PKM) [18], the Basic Markov algorithm [19], Zipf’s cache strategy [20], Zipf’s Markov algorithm [21] and distributed high-speed caching based on spatial and temporal locality (DCST) [14] are all server-side driven models. Studies of all those models show that the algorithms based on user-driven models can improve the cache hit ratio compared to traditional algorithms.

Thus, there is sufficient evidence that users’ behavior is highly correlated with tile access; consequently, mining and using such correlations can help systems prefetch and cache tiles more accurately.

## Related works

Unlike traditional prefetching and caching, a user-driven model fetches tiles and stores them before they are requested based on mining their popularity, or their relationships, or the user’s navigation path—and all those methods are based on users’ behavior.

In client-side prefetching, an application (i.e., a web browser) uses the most recent navigation path of a single individual client to estimate the tiles most likely to be requested next. RAP [17] constructs a heuristic method to predict a client’s next possible movements and then prefetches the tiles that correspond to those movements. RAP assumes that the user’s behavior will not change over a short period (i.e., a user zooming in may continue to zoom-in at the next step). If the user’s behavior changes, RAP launches a brand-new process. RAP uses internal memory to store cached tiles. However, the cache space is very limited; therefore, RAP clears 75% of the cache whenever it runs out of memory.

Server-side prefetching also mines access patterns based on a user’s behavior to find the pertinent relationships among tiles and then to predict and prefetch those objects that are most closely related to the tile being requested. For example, Hilbert Curve-based Prefetching (HCBP) [22] uses the Hilbert Curve to predict the next movement (neighboring tiles) from a user’s current state; PKM [18] also attempts to find the most likely tile among neighboring tiles by computing the transition probabilities between tiles and monitoring the previous *k* movements based on the user’s navigation path; finally PKM uses a Markov Chain to predict the objects to prefetch; Li (2010) [19] and Rui (2012) [21] both used a Markov Chain model to cache an optimum tile. Markov Chain models are constructed by setting the most recently accessed tiles as the initial state and then calculating the probabilities that its neighboring tiles will be requested as a state transition matrix.

However, all the above methods consider only neighboring tiles based on the user's current state—an approach that will introduce the problem of cache annihilation (CA) (i.e., the tiles cached by a one user may be replaced before they are accessed by those cached for another user because of the limited space available in the high-speed cache). Thus, some studies focus on global tiles, attempting to find the optimum choices among all tiles. Shi (2005) [20] calculated the popularity of all tiles and prefetched those with higher probabilities. Rui (2014) [14] also computed the popularity of all tiles and then used the scheme by which the United States Congress is elected to select which tiles to cache: a method called DCST. DCST used a steady-state cache-hit-ratio parameter to limit the tile selection range to save cache space and avoid cache pollution (CP). The authors of [16] proposed Ordinary Least Squares (OLS), a method that used a linear combination of the geographic features and an ordinary least squares regression estimator to predict the user’s future behavior. Furthermore, they also proposed an improved method that used an ANN (Artificial Neural Network) to predict tile popularity [23].

Unfortunately, there are many high access probability hotspot tiles, and it is difficult to find the most appropriate hotspot tiles to cache. The access probability of all data can be computed by considering typical historical access-log information [24] such as the sequence “*GGGGEAFBCDABCDDDCDDDABCDDDCDDD*.” Clearly, D has the highest probability of access in that sequence; C and G are smaller, and E, and F are rare. Thus, based on their access probability, we can prefetch and cache items D and C rather than B in advance when A is requested. However, we can also find that B is typically requested 1 or 2 steps after A is requested. In this case, even though C and D have higher access probabilities than B, B has a closer relationship with A; therefore, B should be prefetched and cached when A is requested.

Meanwhile, simply using tiles’ popularity or their relationships may not accurately represent a user’s behavior in all scenarios. For example, although some of data in the preceding sequence (i.e., E) have closer relationships with G, E does not need to be cached because it is unlikely to be requested again in the future.

Based on the above analyses, we propose a model that uses a brand-new method for combining tiles' popularity and their relationships. It also considers not only global users’ behavior but also global tile relationships. This approach avoids the problems of cache annihilation and cache pollution. The proposed algorithm is named global user-driven prefetching and caching mode (GUDC).

## Global User-driven Models for Tile Prefetching

This model mines the correlation patterns of tiles based on their historical access-log information to prefetch data from the set of hotspot tiles and replace data in the high-speed cache buffer as needed. Investigating a typical example of access such as the sequence shown previously, it is clear that we can make some conclusions about tile correlations:

1. Tiles that are accessed simultaneously will have high correlations;
2. The more often tiles are requested simultaneously during a given timespan, the higher the correlations between them will be;
3. Short access distance intervals (i.e., the steps between two tiles when they are requested chronologically) indicate high correlations.

Thus, the key problem is how to construct a correlation expression model to represent these typical features. For simplicity, we first provide some basic definitions used by the algorithm.

### Trace

For an individual user, there is a sequential navigation path of recorded tiles (the sequence of tiles that were requested by this individual. Similarly, we can sequentially investigate all users’ navigation paths for the entire system; this is called the historical access-log information (or simply referred to as *trace* information).

### Access steps

This value indicates the number of steps between two tiles when they are requested sequentially. For example, if “*ABCD*” are requested sequentially, then the number of access steps between A and B is 1; the number of access steps between A and C is 2; and so on.

### Fixed-access mode

A fixed access mode represents tiles that are usually accessed either sequentially or simultaneously. For example, “AB” and “CD” are two fixed-access modes based on typical historical access-log information [24].

### Matching radius

A large access step value indicates a small correlation; thus, the matching radius denotes the largest access steps between tiles. When tiles' access steps are larger than the matching radius, their correlations are zero.

### Hotspot tiles

Based on Zipf's law, only a small portion of the total tiles will be requested repeatedly [10,11]; these tiles are the hotspot tiles. To avoid invalid prefetching and reduce cache pollution, GUDC prefetches and caches only those hotspot tiles that meet the following restriction [20]:

$$N=K\times h^{1/1-\alpha },$$ (1)

where *K* is the total number of all tiles (the tile set), *N* is the total number of hotspot tiles (a subset of the tile set), *α* is the Zipf distribution parameter and *h* is the steady-state cache hit ratio. The top *N* tiles can be selected as the hotspot tiles set by sorting all tiles based on their popularity from the trace information (or simply by computing the total number of accesses for each tile based on trace information).

In the next two sections, we will propose a simple correlation expression model first, and then, discuss a more complex model based on tiles’ fixed access modes.

### Simple model

To construct a correlation expression model to represent the typical features mentioned above, a **matching degree** model is proposed that follows a conditional prefetching probability algorithm.

Denote *T* = {*t*_1_,*t*_2_,⋯,*t*_*N*_} as the set of all hotspot tiles that will be accessed by all clients in GIS. Each element in *T* is labeled with a natural number [1, *N*], where *N* is the total number of hotspot tiles. Then, let *Q* = (*q*_1_,*q*_2_,⋯,*q*_*L*_) denote all the traces chronologically recorded by system after running for a long period, where *q*_*k*_ ∈ [1,*N*] denotes the label of the *k-*th most-requested tile (i.e., *q*_*k*_ = *i* (*k* = 1,⋯,*L*) indicates that the *k*-th requested tile is *t*_*i*_ (*i* = 1,⋯,*N*)) and *L* is the total number of requests to all hotspot tiles.

If during a certain period *t*_*i*_ is requested and—after *x*-steps—*t*_*j*_ is also requested, then we denote that there is an *x-*step matching from *t*_*i*_ to *t*_*j*_. Then, corresponding matching weights and matching steps can be denoted as *w*_*x*_ and *d*_*x*_, respectively, where *t*_*i*_,*t*_*j*_ ∈ *T* and *d*_*x*_ = *x*, *w*_*x*−1_ > *w*_*x*_ (*i*,*j* ∈ [1,*N*], *i* ≠ *j*). If we denote *n* as the matching radius, then *x* ≤ *n*.

Moreover, for ∀*t*_*i*_ ∈ *T* (*i* ∈ [1,*N*]), we can obtain *G*_*i*_ sub-vectors *Q*_*k*_(*i*) = (*q*_*k*0_,*q*_*k*1_,*q*_*k*2_,⋯,*q*_*kn*_) (*q*_*kx*_ ∈ [1,*N*], *k* ∈ [1,*G*_*i*_], *x* ∈ [0,*n*]), where *q*_*k*0_ = *i*. Obviously, each sub-vector *Q*_*k*_(*i*) indicates which tiles are requested after *t*_*i*_ and indicates their access steps with *t*_*i*_. Thus, the matching degrees from *t*_*i*_ to *t*_*j*_ based on a specific sub-vector *Q*_*k*_(*i*) can be stated as follows:

$$M_{k}(i,j)=\sum_{x=0}^{n}v_{kx}(i,j)w_{x}\;1\leq i,j\leq N,$$ (2)

where

$$v_{kx}(i,j)=\{\begin{array}{l} 1\;q_{kx}^{}=j,j\neq i\\ 0\;Others \end{array}\;j\in [1,N],k\in [1,G_{i}],x\in [0,n],$$ (3)

and their corresponding matching steps *E*_*k*_(*i*,*j*) and matching times *F*_*k*_(*i*,*j*) can be stated as follows:

$$E_{k}(i,j)=\sum_{x=0}^{n}v_{kx}(i,j)d_{x}\;1\leq i,j\leq N$$ (4)

$$F_{k}(i,j)=\sum_{x=0}^{n}v_{kx}(i,j)\;1\leq i,j\leq N.$$ (5)

Considering the full set of sub-vectors $Q(i)=\{Q_{i}(i),Q_{2}(i),\cdots ,Q_{G_{i}}(i)\}$, the total matching degrees *M*(*i*,*j*) from *t*_*i*_ to *t*_*j*_ can be stated as follows:

$$M(i,j)=\sum_{k=1}^{G_{i}}M_{k}(i,j)=\sum_{k=1}^{G_{i}}\sum_{x=0}^{n}v_{kx}(i,j)w_{x}=V(i,j)\cdot W\;1\leq i,j\leq N,$$ (6)

where $V(i,j)=\sum_{k=1}^{G_{i}}V_{k}(i,j)$ and *V*_*k*_(*i*,*j*) = (*v*_*k*0_(*i*,*j*),*v*_*k*1_(*i*,*j*),*v*_*k*2_(*i*,*j*),⋯,*v*_*kn*_(*i*,*j*)) is the matching factor vector based on the sub-vector *Q*_*k*_(*i*), and *W* = (*w*_0_,*w*_1_,*w*_2_,⋯,*w*_*n*_) represents the vector of matching weights. Then,

$$E(i,j)=\sum_{k=1}^{G_{i}}E_{k}(i,j)=V(i,j)\cdot D\;1\leq i,j\leq N\;\mathrm{and}$$ (7)

and

$$F(i,j)=\sum_{k=1}^{G_{i}}F_{k}(i,j)=\sum_{k=1}^{G_{i}}{\left\|V_{k}(i,j)\right\|}_{0}\;1\leq i,j\leq N,$$ (8)

where *D* = (*d*_0_,*d*_1_,*d*_2_,⋯,*d*_*n*_) represents the matching steps vector, and the mathematical expression *V*(*i*,*j*)∙*D* is a dot product between vector *V*(*i*,*j*) and vector *D*.

Eq (6) provides the explicit probability of *t*_*i*_ and *t*_*j*_ being requested simultaneously. Eq (7) yields the access distance between *t*_*i*_ and *t*_*j*_ when they are requested during a short period, and Eq (8) represents the total number of times they are requested simultaneously. Therefore,

$$\vec{P}(i,j)=\frac{M(i,j)}{E(i,j)}\times F(i,j)\;1\leq i,j\leq N$$ (9)

indicates the total prefetch probability for *t*_*j*_ when *t*_*i*_ is requested (in other words, the probability that *t*_*j*_ will be requested next).

A higher matching degree *M*(*i*,*j*) and/or a lower number of matching steps *E*(*i*,*j*) between *t*_*i*_ and *t*_*j*_ denotes a higher probability of *t*_*j*_ being requested either simultaneously or immediately after *t*_*i*_ is requested. In addition, a larger number of matching times *F*(*i*,*j*) can indicate a higher correlation between *t*_*i*_ and *t*_*j*_. Therefore, Eq (9) can satisfy the three obvious conclusions presented previously concerning the correlations of tiles, and it can be used both to compute the correlations among tiles and to predict the next user request based on global users’ behavior. Thus, for ∀*t*_*i*_ ∈ *T*, we have

$$P_{s}(i,T)=(\vec{P}(i,1),\vec{P}(i,2),\cdots ,\vec{P}(i,N)),$$ (10)

from which we can find the element that best predicts the corresponding tiles when *t*_*i*_ is requested.

### Complex model

Furthermore, many studies show that the access to tiles tends to follow a specific path (navigation path) [21,25], consequently, there are many fixed data access modes [26] and we can make predictions using only the last requested tile or we can employ knowledge of these fixed access modes to obtain a more rigorous and accurate forecast. A simple example is shown in Table 1, where the trace comes from typical historical access-log information [24].

**Table 1:** An example of using different conditions to make predictions.

Conditions*	Prediction**
The last requested tile (*t*_*i*_) = *D*	The best choice is *A* or *C or D*
The last two tiles (×*t*_*i*_) = *CD*	The best choice is *A or D*
The last three tiles (××*t*_*i*_) = *BCD*	The best choice is *A or D*
The last four tiles (×××*t*_*i*_) = ABCD^**^	The best choice is *D*

* One or more tiles requested together are considered as a type of fixed access mode.

**Complex conditions can result in a more rigorous and more accurate forecast.

Similarly, for any fixed access mode $A_{k}(i)=(f_{k1}f_{k2}\cdots f_{ka_{k}})$ that ends with *t*_*i*_,

$$\vec{P}(A_{k}(i),j)=\frac{M(A(i),j)}{E(A(i),j)}\times F(A_{k}(i),j)\;1\leq i,j\leq N$$ (11)

yields a conditional prefetch probability for *t*_*j*_ when a series of tiles were requested sequentially based on the fixed access mode *A*_*k*_(*i*) over a short time period, where $f_{ka_{k}}=i$ indicates that the last requested tile is *t*_*i*_, *a*_*kl*_ ∈ [1,*N*] (*l* ∈ [1,*a*_*k*_], *a*_*k*_ ≤ *n*, *k* ∈ [1,*C*_*i*_]), *a*_*k*_ is the length of the fixed access mode *A*_*k*_(*i*), and *C*_*i*_ is the total number of all fixed access modes that end with *t*_*i*_.

Thus, for ∀*t*_*i*_ ∈ *T*, we can obtain a conditional prefetch probability matrix for all tiles based on all possible fixed access modes that end with tile *t*_*i*_. The matrix can be notated as follows:

$$P(A(i),T)=\left[\begin{array}{cccc} \vec{P}(A_{1}(i),1) & \vec{P}(A_{1}(i),2) & \cdots & \vec{P}(A_{1}(i),N)\\ \vec{P}(A_{2}(i),1) & \vec{P}(A_{2}(i),2) & \cdots & \vec{P}(A_{2}(i),N)\\ \vdots & \vdots & \vdots & \vdots \\ \vec{P}(A_{C_{i}}(i),i) & \vec{P}(A_{C_{i}}(i),2) & \cdots & \vec{P}(A_{C_{i}}(i),N) \end{array}\right]$$ (12)

where $A(i)=\{A_{1}(i),A_{2}(i),\cdots ,A_{C_{i}}(i)\}$ represents the set of all fixed access modes that end with *t*_*i*_. Here, *P*_*s*_(*i*,*T*) denotes a specific row of *P*(*A*(*i*),*T*) when its fixed access mode *A*_*k*_(*i*) has only one element(*i*).

### Tile prefetching strategy

As shown in Table 1, the purpose of tile prefetching is to find the best choice to anticipate the user's next movement. Eq (12) shows how to compute the conditional prefetching probabilities for all tiles. Thus, tile prefetching strategies can be stated as follows:

Step 1: Sequentially record the indexes of all tiles requested by all users. After the system has been running for a sufficiently long time, these indexes constitute a historical record denoted as *trace Q*_*all*_.
Step 2: Obtain the set of hotspot tiles set *T* based on *trace Q*_*all*_ using Eq (1). Then, delete all the labels of unpopular tiles from *Q*_*all*_. The result is a hotspot tiles *trace Q*.
Step 3: Sequentially record the index of the most recently requested *n* tiles and denote them as *Q*_*s*_ = (*q*_s1_,*q*_*s*2_,⋯,*q*_*sn*_), where $t_{q_{sn}}$ is the tile being requested.
Step 4: Based on *trace Q*, obtain the fixed access mode set *A*(*q*_*sn*_) and then compute the prefetching probability matrix *P*(*A*(*q*_*sn*_),*T*) for all tiles based on Eq (12).
Step 5: Let $S(q_{sn})=(s_{1}(q_{sn}),s_{2}(q_{sn}),\cdots ,s_{C_{q_{sn}}}(q_{sn}))$ denote the fixed access modes matching indicator of tile $t_{q_{sn}}$ based on the fixed access mode $A(q_{sn})=(A_{1}(q_{sn}),A_{2}(q_{sn}),\cdots ,A_{C_{q_{sn}}}(q_{sn}))$, where $C_{q_{sn}}$ is the number of fixed access modes that end with $t_{q_{sn}}$. Then, *s*_*k*_(*q*_*sn*_) ($k\in [1,C_{q_{sn}}]$) can be shown as follows:

$$s_{k}(q_{sn})=\{\begin{array}{l} 1\;A_{k}(q_{sn})\;is\;a\;sub\;vector\;of\;Q_{s}\\ 0\;Others \end{array}\;k\in [1,C_{q_{sn}}].$$ (13)

Eq (13) indicates that if we can find a certain fixed access mode from the current access states *Q*_s_ then its corresponding fixed access modes matching indicator can be assigned to 1; otherwise, we assign it to zero. Here is a simple example that can be found from the trace discussed above [24]. If we assume that the current access state *Q*_s_ = (*ABCD*), then the tile currently being accessed is *D* (the last element in *Q*_s_). Further, assume that all the fixed access modes of *D* are *A*(*D*) = ((*D*),(*AD*),(*ABD*),(*ABCD*)). Thus, from Eq (13), we obtain *S*(*D*) = (1,0,0,1). Then, using Eq (12) and Eq (13), we can calculate the total conditional prefetching probability, which is stated as follows:

$$P_{f}(q_{sn},T)={\left[\begin{array}{c} \sum_{l=1}^{C_{q_{sn}}}\vec{P}(A_{l}(q_{sn}),1)s_{l}(q_{sn})\\ \sum_{l=1}^{C_{q_{sn}}}\vec{P}(A_{l}(q_{sn}),2)s_{l}(q_{sn})\\ \vdots \\ \sum_{l=1}^{C_{q_{sn}}}\vec{P}(A_{l}(q_{sn}),N)s_{l}(q_{sn}) \end{array}\right]}^{T}=S(q_{sn})\cdot P(A(q_{sn}),T)\;q_{sn}\in [1,N]$$ (14)

Eq (14) shows the total conditional prefetching probabilities based on all the fixed access modes *A*(*q*_*sn*_) that are a dot product between vector *S*(*q*_*sn*_) and matrix *P*(*A*(*q*_*sn*_),*T*). Therefore, the largest element from *P*_*f*_(*q*_*sn*_,*T*) will have the highest probability of being requested next by all users, and we can prefetch its corresponding tile (i.e., if the second item is the largest element in *P*_*f*_(*q*_*sn*_,*T*), then prefetch *t*_2_).

Step 6: Add *Q*_s_ to the end of *Q* and update *Q* and *P*(*A*(*q*_*sn*_),*T*).
Step 7: Repeat Steps 3–7 when the system receives a new request.
Step 8: The tile prefetching algorithm is complete.

Moreover, if we denote the abovementioned method as a 1-step data prefetching strategy, then the *m*-steps data prefetching strategy seeks to obtain *m* tiles that have higher total conditional prefetching probabilities based on Eq (14). Thus, we can use the *m*-steps data prefetching strategy to prefetch more tiles to reduce the computational load and the number of scheduling times.

### Tile replacement strategy

Due to the limited high-speed cache space on servers, another important factor in achieving a high cache-hit rate is to remove the most appropriate data to free cache space on the server using a data replacement strategy. Many classical data replacement algorithms such as FIFO, LRU, and least frequently used (LFU) are widely used in numerous fields [27,28,29] and Google [7], NASA [8] and NGISs [9] have all used the LRU algorithm to achieve good performance in their server systems. The authors of [30] proposed the Lowest Value First Cache Replacement for Geospatial Data (GDLVF), a type of lowest value-first cache replacement algorithm for geospatial data caching that comprehensively considers the influence of many factors, including not only access time and access frequency but also the size of the data size and its location. However, research shows that we can achieve a high hit rate even with a simple LRU technique if we simply group the cache queues [31].

The above analysis is still lacking in that we should consider not only the data itself but also the relationships within the data. Therefore, this article provides a method that comprehensively considers both the global users’ behaviors and all tiles’ relationships based on a unified algorithm model for scheduling tile replacement.

Similar to the tile prefetching strategy, after obtaining *trace Q*, we can schedule tile replacement using the following steps:

Step 1: Sequentially record the indexes of the last *n* cached tiles and denotes the set as *Q*_*c*_ = (*q*_c1_,*q*_*c*2_,⋯,*q*_*cn*_), where $t_{q_{cn}}$ is the tile being prefetched and stored into the high-speed cache.
Step 2: Obviously, the last-cached data must be the last-requested data. Thus, we have $t_{q_{cn}}$ = $t_{q_{sn}}$ and *A*(*q*_*cn*_) = *A*(*q*_*sn*_). Set *P*_*c*_(*A*(*q*_*cn*_),*T*) = *P*(*A*(*q*_*sn*_),*T*)(*q*_*cn*_ ∈ [1,*N*]) and delete the columns from *P*_*c*_(*A*(*q*_*cn*_),*T*) in which the corresponding tiles are not cached (i.e., if tile *t*_*i*_ is not stored in the high-speed cache buffer, then delete the *i*-th column). Thus, the corresponding tiles of *P*_*c*_(*A*(*q*_*cn*_),*T*) are the only tiles stored in the high-speed cache buffer.
Step 3: Similar to Step 5 in the previous section, calculate *S*(*q*_*cn*_) based on tile $t_{q_{cn}}$ and *Q*_*c*_. Then, using Eqs (12) and (13), we can obtain a total conditional caching replacement probability, which can be stated as follows:

$$P_{c}(q_{cn},T)=S(q_{cn})\cdot P_{c}(A(q_{cn}),T)\;q_{cn}\in [1,N].$$ (15)

Eq (15) yields the total conditional caching replacement probabilities based on all fixed access modes; therefore, by finding the smallest one (which has the lowest probability of being requested next by all users) and deleting its corresponding tiles from the high-speed cache buffer, we can save cache space (i.e., if the second item is the smallest element in *P*_*c*_(*q*_*cn*_,*T*), delete *t*_2_ from the cache buffer).

Step 4: Repeat Steps 1–4 when the system prefetches and caches a new tile.
Step 5: The tile caching replacement algorithm is complete.

Using the same logic as the tile prefetching strategy, we can also delete *m* tiles from high-speed cache buffer based on an *m*-step cache replacement strategy.

### Algorithm analysis

A theoretical analysis shows that GUDC must compute the total conditional prefetching probability matrix for all tiles based on the full trace information *Q*. Therefore, GUDC has an initial time complexity of *O*(*N*^3^*L*). Because there are large numbers of datasets (large *N*) and a plethora of trace information, *Q* (large *L*), it is impossible to calculate the conditional prefetching probability matrix in real time; consequently, we must dynamically count and compute matching degrees, matching weights and matching steps for each segment of all the trace information while the system is running. Subsequently, the total conditional prefetching probability matrix can easily be computed by adding all these items based on Eq 6, Eq 7 and Eq 8 after sufficient trace information has been obtained.

## Parameters and Metrics

The proposed method includes two parameters that must be determined: the matching radius *n* and the matching weights vector *W*. The matching radius *n* indicates the correlation depth or the navigation depth, which indicates whether the next *n* movements still have an influence. In this case, Serdar (2012) [17] gives a detailed proposal for navigation depths of approximately 5 to 10; in this case, *n* will vary from 2.5 to 5 considering the symmetry of influence. Therefore, in this article, we can set *n* to 5.

Furthermore, research shows that clients’ requests for tiles satisfy a type of Poisson distribution [32]. If we set the Poisson distribution parameter *λ* = 2*n* (the navigation depth), then we can assume it is a Gaussian distribution, which can be stated as follows:

$$w(x)=\exp(-\frac{{\left(x-\mu \right)}^{2}}{2\sigma^{2}}),$$ (16)

where *μ* can be defined as zero. Then, 95.5% of the effects on a certain tile come from the tiles in which the correlations depth is less than *μ*+1.96*σ* (the Gaussian area between *μ*-1.96*σ* and *μ*+1.96*σ* is 95.5% of the total area). Thus, we can obtain an optimum *σ* = 2.6(*n*/1.96) and using that, we can compute and obtain a matching weights vector *W*. The outputs can then be normalized so that all values fall into the interval [0, 1], satisfying the requirement of the expression model.

In comparison, most similar studies have used the cache-hit ratio (CHR) as the metric for evaluating the prefetch caching performance [23], while some additionally used the average request-response time [13], the effect of network bandwidth [18], refresh time [17] or the byte-hit ratio [8].

The cache-hit ration is defined as the ratio of the number of requests hit in the cache to the total number of cache requests; therefore, obviously, a larger cache-hit ratio reflects a faster average request response time when considering identical systems (i.e., those using identical software and hardware such as Google Earth) and also represents a faster refresh speed or a higher efficient of network bandwidth. Meanwhile, byte-hit ratio is yet another way of expressing CHR. The byte-hit ratio is affected by the sizes of cached tiles or the cache space rather than by the algorithms themselves. Therefore, in this article, we use only one metric—CHR—to evaluate the simulations and experiments.

## Simulations and Experiments

### Simulation design

To illustrate the performance of the proposed algorithm in this paper, we selected our method and some typical methods for comparison:

1. the global user-driven model for tile prefetching proposed in this article, GUDC;
2. The method described in [14], which adopts a fair allocation scheme called the proportional distribution election scheme for the United States Congress and a replication strategy for distributed high-speed caching based on spatial and temporal locality (DCST);
3. The method described in [18], which computes the transition probabilities between tiles and prefetches the most likely neighbors using a type of previous *k* movements algorithm (PKM);
4. The method described in [19], which uses a basic Markov Chain model to cache optimum tiles, called the Basic Markov method (BM);
5. The method described in [20], which calculates the popularity of all tiles and prefetches those tiles with higher probabilities based on Zipf 's law, called the Zipf Law (ZL) method;
6. The method described in [21] that uses a Zipf-like law based on a Markov Chain model to cache optimum tiles, called the Zipf-like Markov method (ZM);

Among these methods, GUDC and DCST can be used to predict requests from multiple users, while all the others are designed to predict only a single user’s behavior. Therefore, comparisons will be made only between GUDC and DCST for multi-user behaviors, and among GUDC, DCST, BM, ZL and ZM based on single user’s behavior.

Based on the analyses discussed above, tile prefetching strategies can be employed on both the server side and client side. Therefore, GUDC will also be implemented as a client-side prefetch method and compared with other client-side algorithms such as (RAP) [17].

Because the Zipf-like distribution parameter *α* can vary significantly, from 0.60 to 1.03 [33], these experiments adopted values of 0.600, 0.750, 0.815, 0.971 and 1.03 for *α* in simulating requests for tiles using GlobeSIGht [11] and to demonstrate the adaptability of the proposed method to the behavior of different users.

For the proposed method, another factor that affects performance is the prefetching steps mentioned at the ends of the previous two sections. Because the best choice for the matching radius is approximately 2.5 to 5, these experiments adopted a prefetching step value of 3, which the means that GUDC will prefetch 3 more tiles whenever a certain tile is requested. We also considered some other choices for the prefetch step value; the corresponding tests are shown below.

Furthermore, to validate the adaptability of the proposed method to different parameters, we conducted some comparative experiments using different matching radii (which vary from 1 to 14), different prefetching steps (which vary from 1 to 10), and different cache replacement strategies (which include the GUDC method, the FIFO algorithm and the LRU algorithm).

### Data sources

The experiments are driven by trace data simulated by accessing SRTM90 (the 90-meter-resolution global terrain data files from the Shuttle Radar Topography Mission) data. All tile requests obey a Zipf-like distribution and are accessed through GlobeSIGht [11], which is an earth observation system similar to Google Earth or NASA World Wind. The requests to servers also obey a Poisson distribution [32].

This study selects some tiles of a certain area of China as the objects to be accessed. The datasets include between 55,242 and 663,552 tiles; however, most requests focus on subsets of the hotspot tiles due to Zipf's law.

The traces include two parts: one is used for training (to compute and find the user-driven model for tile prefetching) and the other is used to test the model validity. Each part of the complete traces includes approximately 2–20 million requests for all the hotspot tiles.

The dataset statistics and the number of requests contained in the traces are summarized in Table 2.

**Table 2:** The datasets parameters used in the experiments.

Parameters	Value
Dataset name	SRTM90
Data size	55242 ~ 663552*
Number of trace	2 for each data size
Trace size	2 million ~ 20 million**

*Based on the Zipf-like laws, only subsets of the hotspot tiles will be requested.

**The traces record only the labels of requested tiles in chronological order.

### The workflow of experiments

The steps to validate the global user-driven cache algorithm were as follows:

1. Using the first portion of the trace information, a correlation matrix and tile popularities were computed;
2. Tiles with higher popularity were prefetched and cached in advance, based on the available cache space;
3. The second part of the trace information was used as a simulation of users’ requests for all hotspot tiles. During the simulation, certain tiles are prefetched and cached; simultaneously, and other tiles in the cache are replaced to save cache space using the various scheduling algorithms mentioned above;
4. For GUDC, the correlation matrix is updated based on the second part of the trace to follow changes in users’ behaviors;
5. For all simulated accesses, the total number of requests hit in the cache was counted, and finally, the cache-hit ratio was calculated.
6. Moreover, the total number of disk accesses was also counted. Then, disk access ratios were computed.

### Experimental results and discussion

There are three kinds of prefetching modes: **multi-user mode**, **single-user mode** and **client-side mode**.

**Multi-user mode** is a global mode that predicts a user's next movement based on the behavior of all users rather than the behavior of a single user.

**Fig 1:**
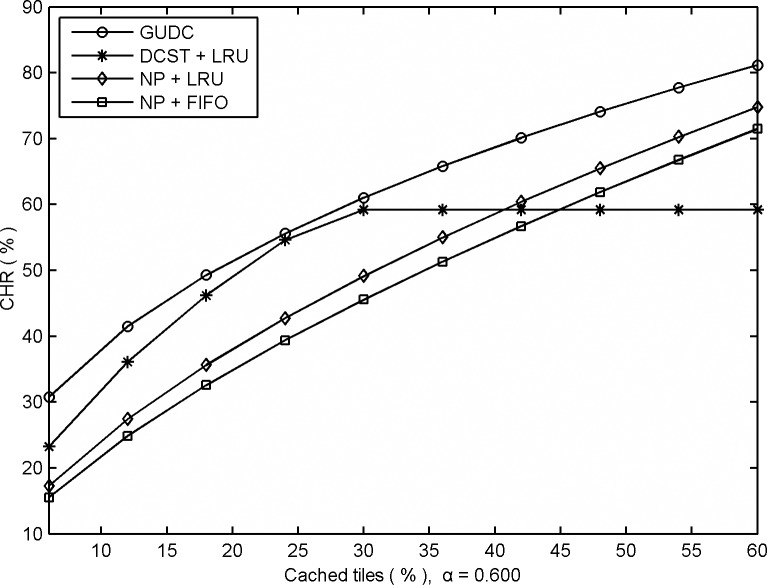
Comparative CHRs obtained from different prefetching algorithms based on multi-user mode.

Fig 1 gives the performances for GUDC, DCST and the No-Prefetching strategy (NP), based on the multi-user mode and measured by the CHR, where the distribution parameter *α* = 0.600. All these algorithms are driven by the global user behavior. GUDC and DCST created an initial cache based on their own algorithm rules. Among these algorithms, GUDC uses the tile replacement strategy proposed in this article to delete tiles from the high-speed cache buffer to save cache space, while DCST, based on its algorithm strategy, uses the LRU strategy to delete tiles from the high-speed cache buffer. NP simply caches the tile being accessed; it never prefetches tiles in advance. NP uses the LRU and FIFO strategies to delete tiles from the high-speed cache buffer.

As shown in Fig 1, the performance of all algorithms improves as the number of cached tiles increases because a greater number of cached tiles increases the possibilities for cache hits. However, due to DCST's cache space saving method, the hit-rate changes only negligibly when the cached tile ratio is above 36% (when *α* = 0.600). In this case, GUDC's performance exceeds that of DCST by approximately 15% when the cached tile ratio is less than 36%, and expands to 27.5% when the cached tile ratio is increased. In addition, GUDC provides obvious performance advantages over the traditional algorithms of FIFO and LRU ranging from 38.7% ~ 51.9% under all experimental conditions.

**Single-user mode** is very different from multi-user mode because it considers only the behavior of one particular user to predict the next movement based on the user’s current observation location.

**Fig 2:**
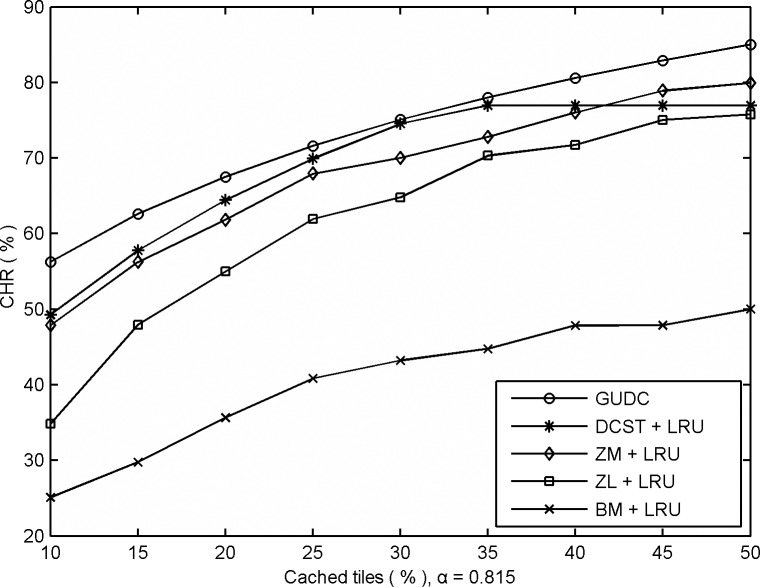
Comparative CHRs obtained from different prefetching algorithms based on single-user mode.

Fig 2 shows the performances of GUDC, DCST, ZM, ZL and BM in single-user mode as measured by the CHR, where the distribution parameter is *α* = 0.815. GUDC and DCST are driven by global user behavior, and the others are driven by a single user’s behavior. All the algorithms in this experiment except GUDC used the LRU cache replacement strategy.

As shown in Fig 2, just as in the first experiment (Fig 1), the performance of all algorithms improves as the number of cached tiles increases. In addition, GUDC achieves a better performance than ZM (by approximately 11.4%), ZL (by approximately 30.7%), and BM (by approximately 110.5%). In this case, DCST achieves a higher CHR than ZM, ZL and BM when the cached tile ratio is less than 42% (when *α* = 0.815), but—for the same reason—its performance advantages diminish as the cached tile ratio increases.

Finally, to investigate GUDC’s performance when implemented as a **client-side prefetching and caching** scheme, a comparative experiment was performed based on client-side cache mode. The performance comparison of different algorithms is shown in Table 3.

**Table 3:** A performance comparison of various algorithms based on the client-side prefetching mode.

Algorithms	Average cache hit ratio (CHR) (%)	Cache space (tiles)	CHR based on normalized cache space (1 tile) (%)
GUDC	56.55%	1000	0.05655
DCST	50.22%	1000	0.05022
ZM	47.83%	1000	0.04783
ZL	34.85%	1000	0.03485
BM	25.08%	1000	0.02508
RAP	50.33%	984	0.05115
PKM	29.41%	1041	0.02825

Due to the limited cache space, algorithms based on client-side cache mode can cache only a limited number of tiles. RAP removes 75% of the oldest tiles from the cache to save cache space; therefore, it has a minimum cache space occupancy ratio. After normalizing cache space to the same benchmark, the performances based on this normalized cache space is computed and shown in the 4th column, which indicates that GUDC achieves the best performance, improving on RAP (which is designed specifically for client-side cache mode) by approximately 10.6%.

**Fig 3:**
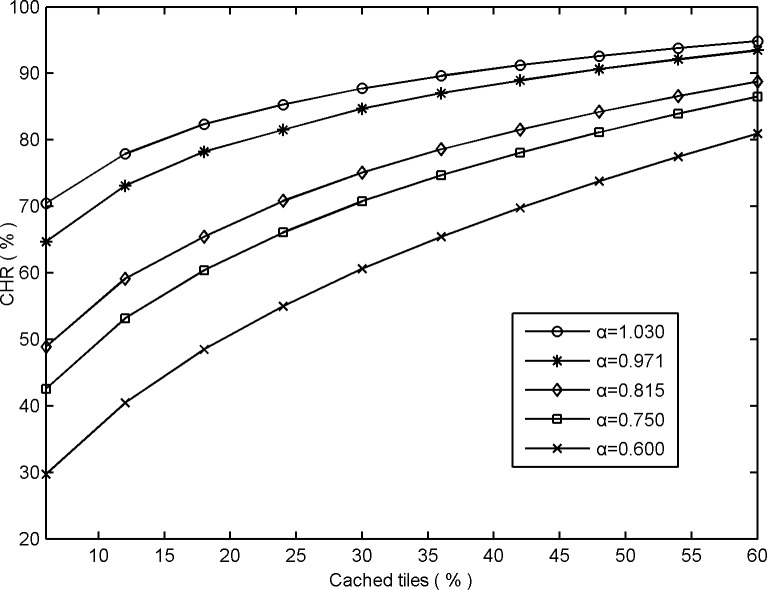
Comparative CHRs obtained from GUDC based on different global user behaviors.

To demonstrate the changes in GUDC's performance based on different Zipf-like distributions, a comparison is shown in Fig 3 in which the distribution parameter *α* changed substantially, from 0.600 to 1.030 (the maximum interval is [0.600–1.03]).

As shown in Fig 3, the performance of GUDC improves as the distribution parameter *α* increases because a larger distribution parameter represents a more concentrated access distribution and, therefore, requires fewer hotspot tiles to be cached. As the distribution parameter increases, the performance improves by an average of approximately 18.5% each time the distribution parameter *α* increases by approximately 10%.

**Fig 4:**
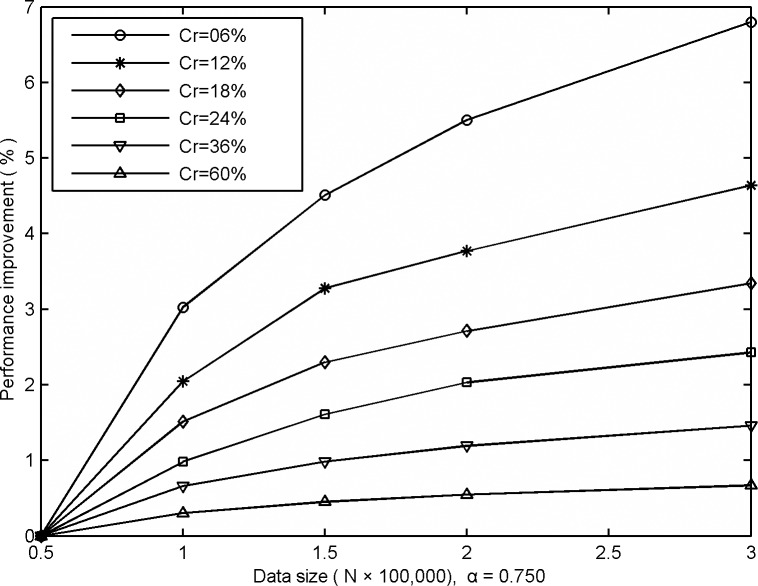
Comparative CHRs obtained from GUDC based on different data sizes.

Furthermore, if we denote (*CHR*_*i*_-*CHR*_1_)/*CHR*_1_ as the performance improvement of GUDC with different data sizes, Fig 4 illustrates the experimental results using different cached tile ratios (*Cr*). In Fig 4, *CHR*_1_ represents the first performance value based on the first data size and *CHR*_*i*_ indicates the performance value resulting from other data sizes.

From Fig 4, it is apparent that the performance improves stably and that a smaller cached tile ratio increase the rate of performance improvement. Through testing and analysis, we found that GUDC can cache more hotspot tiles under the same cached tile ratio as the data size increases. Then, GUDC obtains a higher cache-hit probability, which stably improves the performance as the data size increases. At the same time, comprehensively considering the experimental results in Fig 1 and Fig 3, which show that a smaller cached tile ratio results in lower CHRs as well as that smaller data size result in lower CHRs, it is more difficult to improve GUDC’s performance further because it is very high already.

In contrast, the performance can be improved by increasing the data size, but the same cached tile ratio will lead to a larger cache space demand as well as increased computational complexity because a more massive hotspot dataset must be computed. Thus, in future work, we plan to investigate a way of grouping the tiles to reduce the computational cost while still maintaining GUDC's performance.

**Fig 5:**
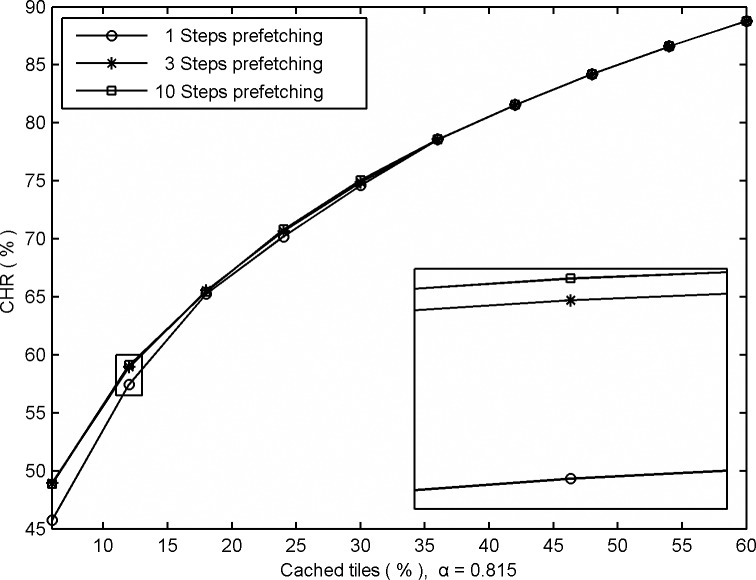
Comparative CHRs obtained from GUDC using different prefetching steps.

Additionally, it is obvious that increasing the number of prefetching steps means more tiles will be cached each time; consequently, we can improve the probabilities of preparing data for the next move in advance. Fig 5 shows the contrast in GUDC's performances with different numbers of prefetching steps (from approximately 1 to 10 tiles, corresponding to 1 to 10 prefetching steps).

Fig 5 shows that the performance can be improved by increasing the number of prefetching steps when the cache space is relatively small, but results in only negligible changes when the number of steps expands beyond 3. The experiment results show that GUDC can accurately represent short-term burst demands among the tiles; therefore, the algorithm achieves better performance using a small number of prefetching steps that reduces the computational complexity. Even a single step is sufficient to obtain a high performance when a large high-speed cache space is available.

**Fig 6:**
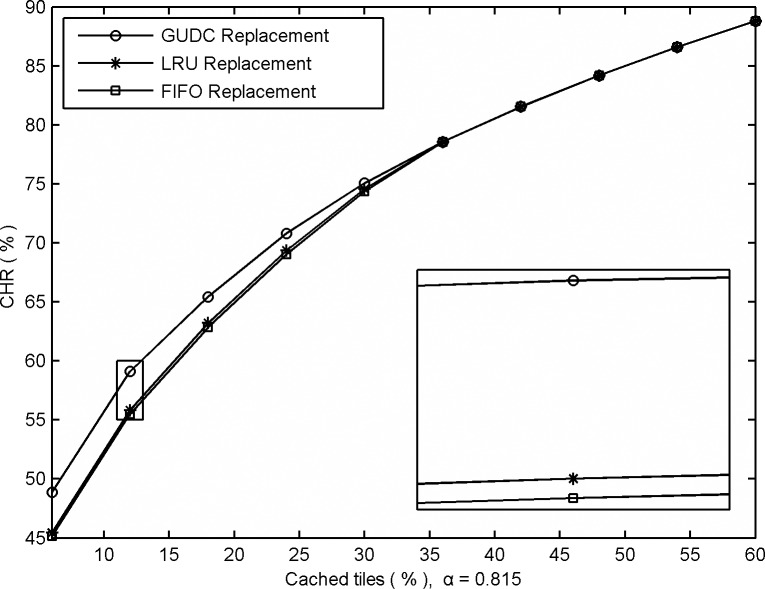
Comparative CHRs obtained from GUDC using different cache replacement strategies.

Tile prefetching and cache replacement are two factors that can help optimize GIS performance. Most algorithms focus only on the prefetching model while using traditional methods to calculate cache replacement. To evaluate the performance of GUDC's cache replacement algorithm, we performed a comparative experiment using the GUDC cache replacement algorithm, the FIFO replacement strategy, and the LRU replacement strategy, as shown in Fig 6.

Similar to the experiment in Fig 5, the performance can be improved by using GUDC cache replacement strategy when the cache space is relatively small. For the same reasons, being able to accurately follow short-term bursts to avoid incorrectly deleting tiles from the cache that will be requested next, has a particularly heavy impact on performance when the cache space is relatively small. From the experimental results shown in Fig 6, we can use the GUDC cache replacement algorithm when the cache space is small or the LRU cache replacement algorithm when the cache space is large to simultaneously reduce computational complexity and obtain high performance.

Because the matching radius, *n*, is another important parameter that can be used to determine the numbers of related tiles, we performed a comparative experiment using different matching radii to compute the matching degrees. This approach results in different total conditional prefetching probability matrix for making predictions and prefetching. A comparison of the results is shown in Table 4, where the distribution parameter *α* = 0.600 and the cached tile ratio was 18%.

**Table 4:** Comparative CHRs and disk access ratio based on different matching radius.

Matching radius (*n*)	Disk access ratio (%)	Average cache hit ratio (CHR) (%)
1	94.77%	48.27%
3	86.92%	48.36%
5	85.23%	48.56%
14	89.62%	48.39%

The 2^nd^ column in Table 4 lists the disk access ratio performance, which is the ratio of the total number of disk accesses (which occurs when the cache is missed and during active prefetching) to the total number of requests from users, and presents the resource cost of the proposed algorithm. The 3rd column shows the CHR performances. Obviously, a lower disk access ratio indicates fewer disk accesses as well as reduced resource consumption. Table 4 sows that the algorithm achieves its best performance based on the designed matching radius (where *n* = 5). Although only small changes occur in the CHRs as the matching radius varies from 1 to 14 (the typical navigation depth is approximately 5 to 10 [17]), the resource cost of the algorithm based on the designed matching radius is the lowest: it reduces the disk access ratio by approximately 4.38%.

**Fig 7:**
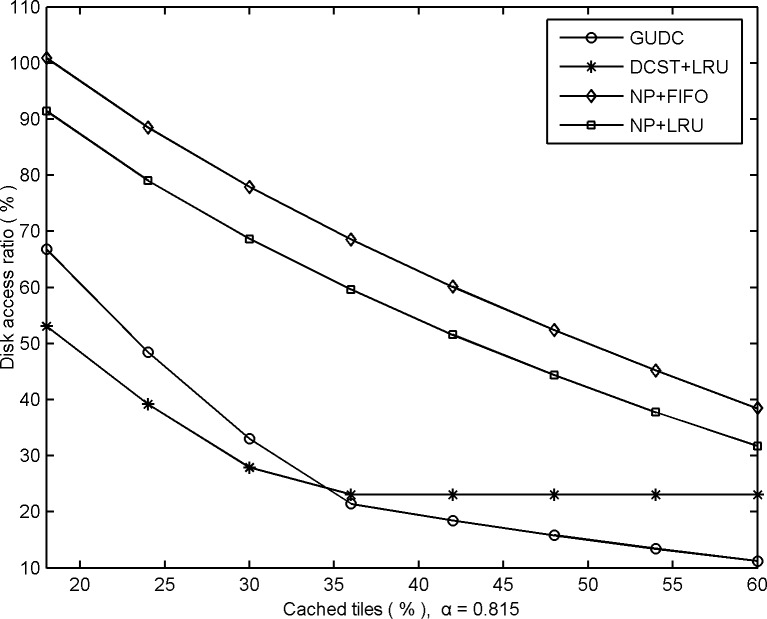
Comparative disk access performance obtained from different algorithms.

Furthermore, to compare the resource costs of different algorithms, a comparative experiment was also conducted using GUDC, DCST and two traditional algorithms. The experimental results are shown in Fig 7.

As Fig 7 shows, GUDC and DCST achieve better disk access ratios than do the two traditional algorithms. Due to limited cache space and maintaining a higher cache hit ratio, GUDC must continually update the cached tiles by prefetching tiles from disk, while DCST simply stores tile being requested into the cache and never actively prefetches data from the disk. Considering the experimental results shown in Fig 1 and Fig 7, the performance gap in disk access ratio can be reduced by increasing the size of the high-speed cache space, which also narrows the CHR performance gap. Moreover, GUDC can continue to reduce disk access ratio by increasing high-speed cache space until, at some point it can be ignored. In contrast, DCST’s disk access ratio cannot be reduced further by increasing the high-speed cache size.

## Conclusions and Future Works

Web geographical information system is a typical service-intensive application which must store massive data into storage nodes and service large numbers of users. Instead of reading tiles from storage in real-time on-the-fly, prefetching and caching tiles that will be requested in the future can reduce the response time of GIS services and substantially improve the quality of service. In server-side cache mode, prefetching and caching tiles can prepare data for servers in advance to reduce the latency of accessing slower disks. In client-side mode, prefetching and caching can be used to reduce the amount of data repeatedly transferred over short periods to save network bandwidth. However, it is difficult to predict the appropriate tiles to prefetch and cache both because of the massive sizes of the datasets as well as the limited space available in high-speed caches. This type of situation requires a more effective method for finding tiles’ inner relationships to trace and predict the next movements of users.

Because access to tiles involves some intrinsic laws that can be mined from historical access records, this study proposed a type of correlation expression method for all tiles that reflects the features of all users’ access behaviors. Then and a global user-driven model for all tile prefetching and cache replacement activities is proposed based on the proposed correlation expression method. This proposed algorithm solves two key problems found in the Zipf’s Markov [21] and DCST [14] algorithms as discussed in the Related Works section of this paper. First, we consider not only tile popularity but also access correlations among tiles. Second, we mine the global access correlations of all tiles through analyzing global user access behaviors to avoid the problem of cache annihilation (CA) and cache pollution (CP). Finally, our algorithm uses the same strategy to perform tile prefetching and cache replacements to realize their coordination. Thus, the proposed integration of the prefetching and caching algorithm can maintain tiles in the cache that are the most likely objects to be requested next while symmetrically removing tiles from the high-speed cache buffer that are the least likely objects to be requested in the future. This global user-driven model is trained (or mined) using historical traces that better describe the relationships among the tiles than the approaches of other proposed algorithms.

In addition, the performance of the proposed method was demonstrated through a series of comparative experiments. The simulation results demonstrate that the proposed algorithm can not only effectively predict the next movements of users in multi-user environments but also adapt to the behaviors of single users. Finally, the proposed GUDC approach can also achieve good results when used for client-side caching.

In total, the method proposed in this study achieves better performance than that of other algorithms in all respects, including approximately a 15.0% ~ 51.9% improvement in multi-user mode, which has recently become one of the most popular research directions, approximately a 11.4% ~ 110.5% improvement in single-user mode, which has attracted many research efforts in the past, and approximately a 10.6% improvement in client-side cache mode, which usually involves limited cache space.

However, the algorithm proposed in this article also requires large trace sizes to achieve a good correlation matrix that closely represents the relationships among tiles. Such large amounts of trace information cannot be guaranteed for new systems. Therefore, in future work, we plan to develop a type of composite method that uses only a user's current status to make predictions when a system is new, but then switches to using traces to mine the relationships among data after sufficient traces are available. Meanwhile, aiming at the drawbacks of conventional distributed computing in computing-intensive application [34], an integrated method that comprehensively considers computational efficiency and access speed also needs to be developed.
